# Network Meta-Analysis of Bevacizumab Gamma Versus Competing Interventions for Treating Neovascular Age-Related Macular Degeneration in the United Kingdom

**DOI:** 10.3390/jmahp13040058

**Published:** 2025-11-19

**Authors:** Maria Lorenzi, Stephen Ebohon, Jennifer Kissner, Jedd Comiskey, Mayke Paap, Christine Bouchet, Andy Garnham, Erika Wissinger

**Affiliations:** 1 Cencora Inc. US, 1 West First Avenue, Conshohocken, PA 19428, USA; maria.lorenzi@cencora.com; 2 Cencora Inc. UK, 10 Fetter Ln, London EC4A 1BR, UK; stephen.ebohon@cencora.com (S.E.); andygarnham@neonavitas.com (A.G.); 3 Outlook Therapeutics Inc., 111 Wood Ave, Iselin, NJ 08830, USA; jeddcomiskey@outlooktherapeutics.com (J.C.); christinebouchet@outlooktherapeutics.com (C.B.)

**Keywords:** nAMD, anti-VEGF, indirect comparison, visual acuity

## Abstract

This study aimed to determine the relative efficacy of bevacizumab gamma (an ophthalmic formulation of bevacizumab) versus alternative interventions relevant to the treatment of neovascular age-related macular degeneration (nAMD) in the United Kingdom (UK) via a systematic literature review (SLR) and network meta-analysis (NMA). An SLR was conducted to identify randomized controlled trials (RCTs) of anti-vascular endothelial growth factor (anti-VEGF) therapies for the treatment of nAMD in adult patients relevant to the UK context. The included anti-VEGF treatments were ranibizumab, aflibercept, faricimab, and bevacizumab gamma. Bayesian NMA models were used to estimate relative efficacy in terms of change from baseline (CFB) in best-corrected visual acuity (BCVA) at 12 months, the proportion of patients gaining 15 or more letters at 12 months, and the proportion of patients losing less than 15 letters at 12 months. Twenty-two relevant RCTs were included in the NMA. At 12 months, all anti-VEGF treatments were similarly efficacious to ranibizumab 0.5 mg every four weeks (Q4W) in terms of CFB in BCVA, the proportion of patients gaining 15 or more letters, and the proportion of patients losing less than 15 letters (except for ranibizumab 0.5 mg every 12 weeks [Q12W] and ranibizumab 0.5 mg pro re nata [PRN]). Bevacizumab gamma provided similar improvements in visual acuity to other anti-VEGF treatments.

## 1. Introduction

Age-related macular degeneration (AMD) is a leading cause of visual impairment in the UK [1,2]. However, there is a paucity of recent epidemiologic data for late-stage AMD—according to a meta-analysis of 2007–2009 population data, the prevalence of late-stage AMD among individuals aged 50 and older was approximately 2.4% of the population, emphasizing its considerable public health burden [3]. Among the two forms of late-stage AMD, the neovascular subtype (nAMD) is particularly aggressive, characterized by abnormal blood vessel growth under the retina and often leading to rapid central vision loss. The emergence of anti-vascular endothelial growth factor (anti-VEGF) therapies has significantly advanced the management of nAMD, providing new opportunities in halting disease progression. Although currently available anti-VEGF treatments are generally regarded as having comparable efficacy, variations in treatment regimens may influence clinical outcomes [4,5,6]. Notably, studies have suggested that treat-and-extend (TREX) regimens have been associated with better visual acuity improvements compared to fixed monthly or pro re nata (PRN) dosing, particularly for ranibizumab [7]. In an effort to optimize treatment accessibility, repackaged bevacizumab has been used off-label to treat nAMD; however, handling and distribution processes may contribute to variable efficacy, complicating its use in clinical practice [7]. These challenges highlight the importance of evaluating ophthalmic formulations as a potential alternative. Bevacizumab gamma (Lytenava™) is an ophthalmic formulation of bevacizumab, that, in the pivotal Phase 3 NORSE TWO trial, demonstrated favorable efficacy, with 41.7% of patients gaining 15 or more letters of vision at month 11, and a mean increase in best-corrected visual acuity (BCVA) of 11.2 letters with a standard deviation (SD) of 12.19 [8]. While off-label bevacizumab has been compared to other anti-VEGF regimens, no indirect treatment comparison has been undertaken to compare bevacizumab gamma to other anti-VEGF regimens [5,6]. The objective of this study was to conduct a systematic literature review (SLR) and network meta-analysis (NMA) comparing bevacizumab gamma to other anti-VEGF agents relevant to managing nAMD among patients in the United Kingdom (UK), with particular attention to variations in dosing strategies.

## 2. Materials and Methods

### 2.1. Materials and Methods: Systematic Literature Review

The SLR was conducted in accordance with guidance from the Preferred Reporting Items for Systematic Reviews and Meta-Analyses (PRISMA) statement and health technology assessment agencies, such as the UK National Institute for Health and Care Excellence (NICE), and followed a pre-specified SLR protocol. Database searches were conducted in Embase, MEDLINE, and CENTRAL via Ovid on 25 October 2022 and updated on 30 January 2024. The search used medical subject headings (MeSH) terms, validated study design filters, and free-text terms specific to the population, interventions, and study designs of interest (Search strategy in Tables S1 and S2). Population, intervention, comparison, outcome measures, and study design (PICOS) criteria were used for study selection (Table 1). The PICOS criteria specified the inclusion of RCTs enrolling adult (age ≥ 18 years) patients with nAMD. The following interventions were included in the search strategy: bevacizumab, faricimab, aflibercept, conbercept, ranibizumab, brolucizumab, and pegaptanib.

Title/abstract and full-text screening were conducted by two independent reviewers; a third reviewer resolved any discrepancies. Data extraction and risk of bias (RoB) assessment using the RoB 1 tool [9] was conducted by one reviewer, and full validation was performed by a second reviewer. For the change from baseline (CFB) in BCVA, for study arms with missing information on variability, SD was imputed by calculating the mean SD from study arms with reported measures of variability. To focus the NMA on approved interventions and regimens relevant to current clinical practice in the UK health system, studies evaluating bevacizumab gamma, faricimab, aflibercept, and ranibizumab were selected for inclusion in the analysis.

### 2.2. Materials and Methods: Network Meta-Analysis

In the absence of data from head-to-head clinical trials, NMA is a viable method to estimate the relative efficacy of interventions that have not been directly compared to each other [10,11,12].

A Bayesian NMA was conducted; ranibizumab 0.5 mg every four weeks (Q4W) was used as the common comparator/reference node. Dichotomous variables were synthesized using a standard model with a binomial likelihood and logit function; for continuous outcomes, mean CFB was analyzed using an identity link and normal likelihood. For all models, non-informative normal priors N (0, 100^2^) were used for treatment effects and study-specific intercepts. Both fixed-effects and random-effects models were fitted; models were compared using the deviance information criterion (DIC) and the total residual deviance. Random-effects model results are presented in this manuscript. Sensitivity analyses were conducted for each analysis, excluding studies with 100% Asian patient populations (DRAGON, Haga 2018) from the network to assess the impact of these studies on the overall results [13,14]. Surface area under the cumulative ranking curve (SUCRA) values were calculated for each treatment in each analysis conducted.

All analyses were performed using R version 4.4.0. Model parameters were estimated using a Hamiltonian Markov Chain Monte Carlo algorithm implemented in the multinma package [15]. The results comprise 50,000 samples from the posterior distribution of each model, after 50,000 “burn-in” iterations.

## 3. Results

### 3.1. Results: Systematic Literature Review

**Figure 1:**
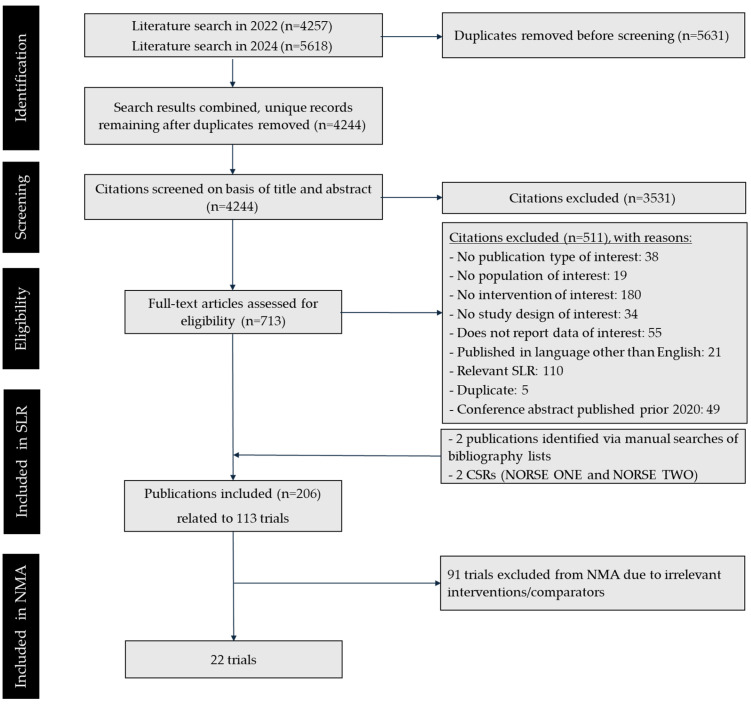
PRISMA diagram. Key: CSR—clinical study report; SLR—systematic literature review.

After removing duplicates, 4244 unique citations were screened at the title/abstract level, with 3531 excluded at this stage. The full-text publications of the remaining 713 abstracts were retrieved and screened at the full-text level. Ultimately, 206 publications pertaining to 113 trials met the inclusion criteria and were included in the SLR. Of these studies, 22 were identified as relevant to the treatment of nAMD in the UK and were subsequently included in the feasibility assessment for an NMA (Table 2). The PRISMA diagram representing the flow of study selection is presented in Figure 1. Results of RoB assessment are presented in Figure S1.

To assess heterogeneity across the evidence base, the 22 included studies were compared in terms of reported baseline patient characteristics, reported outcomes, and study characteristics. Age is a recognized prognostic factor for macular degeneration; however, the included studies exhibited reasonably similar patient mean ages, spanning from 66.6 years to 79 years. Body mass index (BMI) is considered a modifiable risk factor for nAMD [34]; however, its assessment for the included patient populations was hindered by insufficient reporting across studies. The proportion of white patients included varied between 68.8% to 97.8%. Caucasians typically face an increased risk of developing nAMD, and some studies suggest that ranibizumab may be less effective in non-white patients compared to white patients [35,36]. Additionally, the types of choroidal neovascularization (CNV) (occult, predominantly classic, and minimally classic) differed among patient populations across the trials, and CNV has been documented to impact visual and anatomical outcomes in eyes with nAMD following anti-VEGF treatment [37]. Reporting, however, was inconsistent: some studies aggregated categories, whilst others distinguished subtypes and, in some cases, added classic as an additional category. Such variability limited the assessment of lesion subtype as an effect modifier. All but one (Mori 2017) of the 22 included studies reported the proportion of patients gaining at least 15 letters between 11 and 12 months [25]. All studies except CANTREAT and Chan 2015 reported CFB in BCVA at a time point between 11 and 12 months [18,20].

### 3.2. Results: Network Meta-Analysis

**Figure 2:**
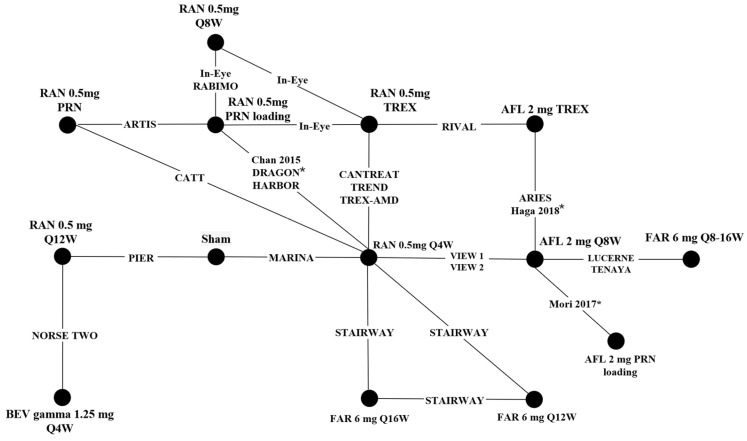
Network of evidence. Key: AFL—aflibercept; BEV—bevacizumab; FAR—faricimab; PRN—pro re nata; RAN—ranibizumab; TREX—treat and extend. * Studies in 100% Asian patients are indicated with an asterisk. Full list of studies included in Figure 2: ARIES [16]; ARTIS [17]; CANTREAT [18]; CATT [19]; Chan 2015 [20]; DRAGON [14]; HARBOR [21]; Haga 2018 [13]; In-Eye [22]; LUCERNE [23]; MARINA [24]; Mori 2017 [25]; NORSE TWO [26]; PIER [27]; RABIMO [28]; RIVAL [29]; STAIRWAY [30]; TENAYA [23]; TREND [31]; TREX-AMD [32]; VIEW 1 [33]; VIEW 2 [33].

The 22 included studies form a connected network of evidence, shown in Figure 2.

Models were evaluated using the DIC to facilitate comparison, while their goodness-of-fit was assessed through an analysis of residual deviance. Since the DIC values did not differ meaningfully between fixed-effects and random-effects models in any analysis, results using the random effects model are presented throughout.

#### 3.2.1. BCVA at 12 Months

**Figure 3:**
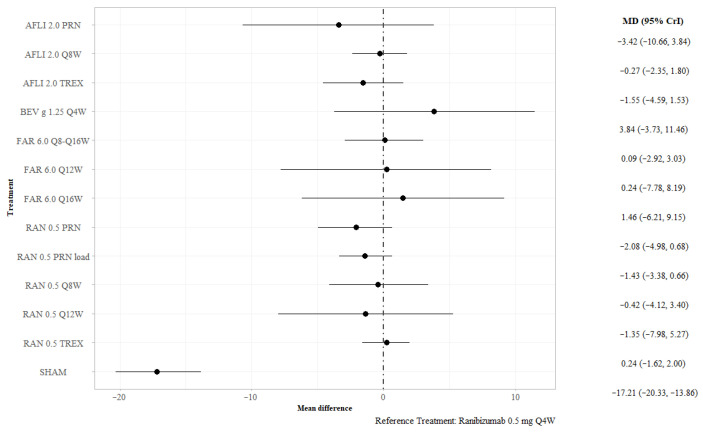
BCVA at 12 months, all interventions versus ranibizumab 0.5 mg Q4W. Key: AFL—aflibercept; BEV—bevacizumab; FAR—faricimab; PRN—pro re nata; RAN—ranibizumab; TREX—treat and extend.

Trial-level estimates of mean change in BCVA at 12 months were synthesized (Table S3). Under the random-effects model, no active interventions differed statistically from ranibizumab 0.5 mg QW4 for CFB in BCVA at 12 months. Sham treatment was associated with a statistically smaller mean difference in BCVA at 12 months than ranibizumab 0.5 mg QW4 (MD −17.21; 95% credible interval [CrI] −20.33, −13.86) (Figure 3). Ranibizumab 0.5 mg every 12 weeks (Q12W) was less efficacious than ranibizumab 0.5 mg Q4W for this outcome (MD −5.23; 95% CrI −9.47, −0.91); no other statistical differences between treatments were found (Table S4—league table). The surface under the cumulative ranking curve (SUCRA) for bevacizumab gamma 1.25 mg Q4W was 0.863, the highest among all included treatments (Table S8); this indicates a strong probability of being the best or among the best treatment options available for this outcome. In the sensitivity analyses, excluding studies with 100% Asian populations yielded results consistent with the base case (Table S7).

#### 3.2.2. Proportion of Patients Gaining at Least 15 Letters at 12 Months

**Figure 4:**
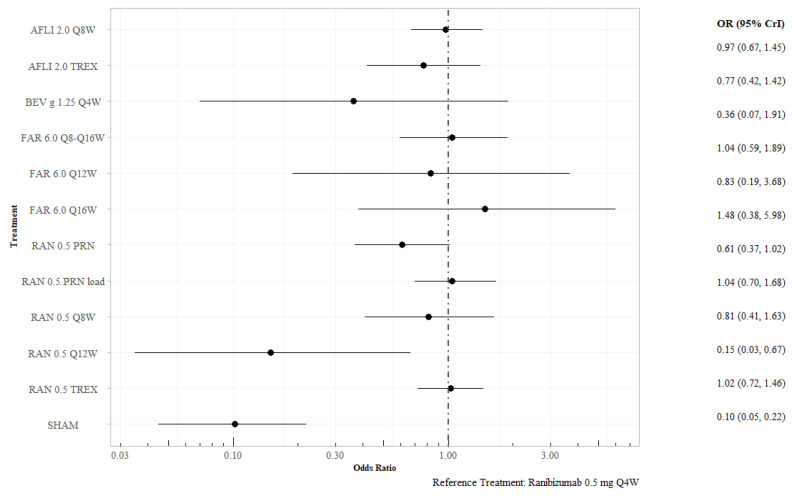
Proportion of patients gaining 15 or more letters at 12 months, all interventions versus ranibizumab 0.5 mg Q4W. Key: AFL—aflibercept; BEV—bevacizumab; FAR—faricimab; PRN—pro re nata; RAN—ranibizumab; TREX—treat and extend.

Twenty-one included trials reported the proportion of patients gaining at least 15 BCVA letters at 12 months. Ranibizumab 0.5 mg Q12W (odds ratio [OR] 0.15; 95% CrI 0.04–0.64), ranibizumab 0.5 mg PRN (OR 0.59; 95% CrI 0.35–0.95), and sham treatment (OR 0.10; 95% CrI 0.05–0.22) were statistically less efficacious than ranibizumab 0.5 mg Q4W (Figure 4). Ranibizumab 0.5 mg Q12W was the least efficacious of the active interventions and was statistically associated with lower odds of gaining at least 15 letters compared to aflibercept 2.0 mg every eight weeks (Q8W), aflibercept 2.0 mg Q8W treat-and-extend (TREX), faricimab 6.0 mg every 16 weeks (Q16W), bevacizumab gamma 1.25 mg QW4, and ranibizumab 0.5 mg PRN with a loading dose (Table S5). Sensitivity analysis for this outcome showed that excluding studies with 100% Asian populations did not alter the conclusions (Table S7).

#### 3.2.3. Proportion of Patients Losing Fewer than 15 Letters at 12 Months

**Figure 5:**
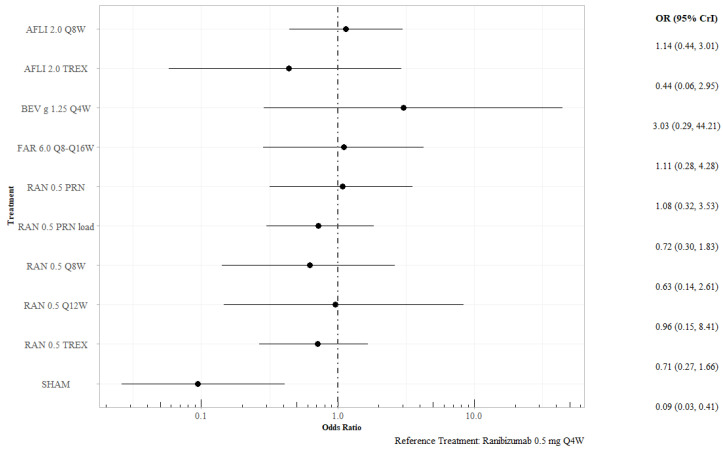
Proportion of patients losing fewer than 15 letters at 12 months, all interventions versus ranibizumab 0.5 mg Q4W. Key: AFL—aflibercept; BEV—bevacizumab; FAR—faricimab; PRN—pro re nata; RAN—ranibizumab; TREX—treat and extend.

At 12 months, sham was associated with significantly lower odds of losing fewer than 15 letters than ranibizumab 0.5 mg Q4W (OR 0.09; 95% CrI 0.03–0.41), with no other differences observed between ranibizumab 0.5 mg Q4W and the other active interventions (Figure 5). Excluding studies with 100% Asian patient populations did not alter these conclusions (Table S7). The SUCRA for bevacizumab gamma 1.25 mg QW4 was 0.879.

## 4. Discussion

The objective of this study was to evaluate the relative efficacy of anti-VEGF regimens used for the treatment of patients with nAMD in UK real-world clinical practice. Relevant data were obtained from an SLR of clinical evidence, which identified 22 unique RCTs that met the eligibility criteria. After conducting a feasibility assessment, these trials were synthesized using a Bayesian NMA to estimate the relative treatment effect of included interventions in terms of CFB in BCVA at 12 months, and the proportion of patients gaining at least 15 letters at 12 months. For the NORSE TWO trial (evaluating bevacizumab gamma), 11-month data were compared to 12-month data from other studies in the majority of cases, due to the study design of NORSE TWO. Definitions and measurements were standardized across trials, enabling robust comparisons.

The patient populations across the included studies were broadly similar in terms of age, sex, type of CNV, and race distribution. However, BMI, despite being a modifiable risk factor for nAMD, could not be evaluated due to lack of reporting across the trials [34]. Additionally, baseline retinal thickness measurements varied among the studies due to the use of different techniques and definitions for assessing thickness. Given the limited epidemiological data available for patients with nAMD in the UK who are treated with anti-VEGFs, it is challenging to assess the generalizability of the patient populations included in the NMA to real-world patient populations. However, based on a recent National Ophthalmology Database Audit, the majority of patients starting treatment in the 2021 National Health Service year were female and Caucasian, with a median age of 80.5 years at the start of treatment, which is broadly aligned with the populations of the included trials [38].

At 12 months, no statistical difference was found among active interventions in terms of change in BCVA, although bevacizumab gamma exhibited the highest numerical mean difference in BCVA and the highest SUCRA value. Regarding the proportion of patients gaining at least 15 letters, bevacizumab gamma was not statistically different from other active interventions, with the exception of ranibizumab 0.5 Q12W. These findings align with recent NMAs that have found similar efficacy among anti-VEGF regimens [4,5,6].

Strengths of this analysis include the derivation of an evidence base identified via a comprehensive SLR. Furthermore, the evidence base available for the NMA consisted of a connected network of RCTs, allowing for an anchored indirect comparison of the interventions of interest. The Bayesian framework allowed for a thorough assessment of comparative efficacy, leveraging all available data. A sensitivity analysis removing two studies conducted in 100% Asian populations (DRAGON, Haga 2018) was performed; the results confirmed those of the base case analysis [13,14]. These results are consistent with those of Zhao et al., who conducted a comprehensive NMA and concluded that, when applying the same treatment strategy, there is no significant difference in efficacy or safety among ranibizumab, bevacizumab, and aflibercept [39]. Furthermore, the conclusions of the current NMA align with previous studies such as CATT [19] and IVAN [40], which demonstrated non-inferiority of bevacizumab to ranibizumab when used in similar dosing regimens. However, these studies used a bevacizumab solution intended for oncologic use and repackaged for intravitreal injections, which may result in variability in the repackaged bevacizumab [41], whereas bevacizumab gamma has been specifically manufactured, formulated, and packaged according to the standards of ophthalmic solutions.

There are several limitations that should be considered when interpreting the results of this NMA. The connection through the sham treatment is not particularly robust, as both included trials assessed a sham arm with a high proportion of patients who had prior anti-VEGF exposure, which could serve as an effect modifier and impact the relative treatment effect estimates. In addition, the low event rates observed in the sham arms led to unstable estimates of the relative treatment effects of bevacizumab gamma compared to other competing interventions. Furthermore, the study design of NORSE TWO provided data at 11 months, compared to 12-month data availability for other trials; the two time points were deemed comparable in order to obtain estimates of relative treatment effects, as most included trials incorporated a visit window of +/−4 weeks into reported 12-month data. Lastly, although standard deviations were missing in 12 of 22 studies and required imputation, this was undertaken in accordance with NICE recommendations to ensure comprehensive network connectivity. While this represents a limitation, this approach was essential to preserve the integrity of the evidence base and to ensure the analysis could be robustly conducted.

## 5. Conclusions

In conclusion, bevacizumab gamma demonstrated efficacy in managing nAMD across multiple outcomes and provided similar improvements in visual acuity to other anti-VEGF treatments relevant to the UK healthcare system setting. The robustness of these findings and the strength of the conclusions would benefit from additional direct evidence comparing bevacizumab gamma with the other active interventions in the network.

## Figures and Tables

**Table 1:** PICOS.

Domain	Inclusion Criteria	Exclusion Criteria
Population	Adult (≥18 years old) patients with nAMD	Any divergent population, such as: Patients with other diseases Mixed populations of patients (e.g., all AMD) without stratified outcomes data for the population of interest Pediatric populations
Interventions	Bevacizumab Faricimab Aflibercept Conbercept Ranibizumab Brolucizumab Pegaptanib Biosimilar formulations of the above interventions of interest All potential therapeutic regimens were evaluated: Fixed interval: injections given according to a pre-specified schedule (e.g., Q4W, Q8W, Q12W, Q16W) PRN (pro re nata): injections given as needed, defined a priori in the protocol of the specific study PRN and extend (pro re nata and extend): PRN with the opportunity to extend the interval between assessments TREX (treat-and-extend): treatment with the flexibility to adjust the timing between injections (e.g., +2-week adjustment, −2-week adjustment)	Any other intervention not listed in the Inclusion column
Comparators	Any other interventions of interest Placebo/sham Standard of care/observation Any other treatment that provides the necessary link(s) to conduct an indirect comparison (e.g., to create a connected network of treatments for analyses)	Any other comparator not listed in the Inclusion column
Outcomes	Efficacy, e.g., BCVA (mean change from baseline, proportion of patients who achieve threshold change) Visual acuity Snellen equivalent Central foveal thickness Safety, e.g., Proportion of patients who experienced any AE Proportion of patients who experienced an ocular AE Proportion of patients who experienced a serious AE Proportion of patients who discontinued treatment due to AE	Any outcomes not listed in the Inclusion column
Study design	RCTs Existing published SLRs/meta-analyses of RCT data were included for manual bibliography checks	Any other study design not listed in the Inclusion column
Time	No date restriction for full-text articles; 2020–2024 for conference abstracts	Conference abstracts published before 2020
Language	English language publications	Publications in languages other than English

Key: AE—adverse event; BCVA—best-corrected visual acuity; nAMD—neovascular age-related macular degeneration; Q4W—every four weeks; Q8W—every eight weeks; Q12W—every twelve weeks; Q16W—every sixteen weeks; RCT—randomized controlled trial; SLR—systematic literature review.

**Table 2:** Studies included in network meta-analysis.

Study/Reference	Treatment	Total N	Age, Mean (SD)	Proportion White Pts	CNV Type, %
ARIES [16]	AFLI 2.0 mg TREX	106	75.5 (9)	NR	NR
	AFLI 2.0 mg Q8W TREX	104	76.6 (8.7)	NR	NR
ARTIS [17]	RAN 0.5 mg PRN	45	69.7 (8.6)	NR	NR
	RAN 0.5 mg PRN loading	49	70 (8.8)	NR	NR
CANTREAT [18]	RAN 0.5 mg TREX	287	78.9 (7.7)	95.5	NR
	RAN 0.5 mg Q4W	293	78.7 (8)	93.2	NR
CATT [19]	RAN 0.5 mg Q4W	301	79.2 (7.4)	98.7	NR
	RAN 0.5 mg PRN	298	78.4 (7.8)	99.3	NR
Chan 2015 [20]	RAN 0.5 mg Q4W	6	82 (6.2)	NR	NR
	RAN 0.5 mg PRN loading	7	84 (6.0)	NR	NR
DRAGON [14]	RAN 0.5 mg Q4W	167	65.6 (8.43)	0	PC 18; MC 9; OC 65.3; Other 7.8
	RAN 0.5 mg PRN loading	166	66.8 (8.31)	0	PC 17.5; MC 7.8; OC 66.9; Other 10.2
HARBOR [21]	RAN 0.5 mg Q4W	275	78.8 (8.4)	964.	PC 15.3; MC 46.2; OC 38.5;
	RAN 0.5 mg PRN loading	275	78.5 (8.3)	97.5	PC 17.1; MC 46.5; OC 36.4;
Haga 2018 [13]	AFLI 2.0 mg TREX	21	75.5 (6.7)	0	NR
	AFLI 2.0 mg Q8W	20	78.5 (8.1)	0	NR
In-Eye [22]	RAN 0.5 mg Q8W	92	76.9 (7.4)	NR	NR
	RAN 0.5 mg TREX	88	77.8 (7.4)	NR	NR
	RAN 0.5 mg PRN loading	90	77.5 (8.2)	NR	NR
LUCERNE [23]	FAR 6.0 mg Q8-Q16W	331	74.8 (8.4)	84	PC 2; MC 9; OC 52; Cl 30
	AFLI 2.0 mg Q8W	327	76.1 (8.6)	83	PC 5; MC 9; OC 43; Cl 33
MARINA [24]	RAN 0.5 mg Q4W	240	77 (8)	96.7	PC 0; MC 37.9; OC 62.1; Other 0
	SHAM	238	77 (7)	97.1	PC 0; MC 36.6; OC 63.4; Other 0
Mori 2017 [25]	AFLI 2.0 mg PRN	30	76.5 (NR)	NR	NR
	AFLI 2.0 mg Q8W	28	72.8 (NR)	NR	NR
NORSE TWO [26]	BEV gamma 1.25 mg Q4W	113	78.8 (8.3)	97.3	NR
	RAN 0.5 mg Q12W	115	79.1 (8.5)	98.3	NR
PIER [27]	SHAM	63	77.8 (7.1)	93.7	PC 22.2; MC 46; OC 31.7; Other 0
	RAN 0.5 mg Q12W	61	78.8 (7.9)	91.8	PC 21.3; MC 29.5; OC 49.2; Other 0
RABIMO [28]	RAN 0.5 mg Q8W	20	79 *	NR	NR
	RAN 0.5 mg PRN loading	20	81 *	NR	NR
RIVAL [29]	RAN 0.5 mg TREX	142	76.6 (8.5)	93	PC 15; MC/OC 84; Other 1
	AFLI 2.0 mg TREX	139	78.7 (7.5)	93.5	PC 19; MC/OC 80; Other 1
STAIRWAY [30]	RAN 0.5 mg Q4W	16	77.3 (10.3)	100	Cl/OC 37.5; Cl 12.5; OC 50
	FAR 6.0 mg Q12W	24	80.3 (7.2)	95.8	Cl/OC 37.5; Cl 0; OC 62.5
	FAR 6.0 mg Q16W	31	77.7 (8.4)	96.8	Cl/OC 29; Cl 6.5; OC 64.5
TENAYA [23]	FAR 6.0 mg Q8-Q16W	334	75.9 (8.6)	91	PC 5; MC 10; OC 53; Cl 25
	AFLI 2.0 mg Q8W	337	76.7 (8.8)	90	PC 6; MC 9; OC 52; Cl 22
TREND [31]	RAN 0.5 mg TREX	323	75.3 (8.61)	91.6	PC 8.4; MC 10.2; OC 42.7; Cl 26.6; Other 12.1
	RAN 0.5 mg Q4W	327	75.2 (8.13)	92	PC 7; MC 3.4; OC 52.3; Cl 26.9; Other 10.5
TREX-AMD [32]	RAN 0.5 mg Q4W	20	NR	NR	NR
	RAN 0.5 mg TREX	40	NR	NR	NR
VIEW 1 [33]	RAN 0.5 mg Q4W	304	78.2 (7.6)	97.4	PC 27; MC 33.2; OC 37.8;
	AFLI 2.0 mg Q8W	301	77.9 (8.4)	95.3	PC 23.6; MC 36.5; OC 39.2;
VIEW 2 [33]	RAN 0.5 mg Q4W	291	73 (9)	73.2	PC 24.1; MC 35.7; OC 39.9;
	AFLI 2.0 mg Q8W	306	73.8 (8.6)	70.9	PC 28.8; MC 34.6; OC 35.9;

Key: AFLI—aflibercept; BEV—bevacizumab; Cl—classic; FAR—faricimab; MC—minimally classic; NR—not reported; OC—occult; PRN—pro re nata; PC—predominantly classic; RAN—ranibizumab; TREX—treat and extend. * median.

## Data Availability

As a network meta-analysis, no new data were created or analyzed in this study.

## Supplementary Materials

The following supporting information can be downloaded at: https://www.mdpi.com/article/10.3390/jmahp13040058/s1, Table S1: Search strategy—original search; Table S2: Search strategy—search update; Figure S1: RoB assessment results; Table S3: Trial-level input data for change from baseline in BCVA at 12 months; Table S4: Change from baseline in BCVA at 12 months; mean differences (95% CrI) for all possible pairs of interventions; Table S5: Proportion of patients gaining at least 15 letters at 12 months; OR (95% CrI) for all possible pairs of interventions; Table S6: Proportion of patients losing fewer than 15 letters at 12 months; OR (95% CrI) for all possible pairs of interventions; Table S7: Comparison of base case and sensitivity analysis removing studies with 100% Asian patients; Table S8: SUCRA rankings of treatments in each analysis.

## Abbreviations

The following abbreviations are used in this manuscript:

AFL	aflibercept
Anti-VEGF	anti-vascular endothelial growth factor
BCVA	best-corrected visual acuity
BEV	bevacizumab
BMI	body mass index
CFB	change from baseline
CNV	choroidal neovascularization
CrI	credible interval
DIC	deviance information criterion
FAR	faricimab
MAIC	matching-adjusted indirect comparison
MeSH	Medical Subject Headings
nAMD	neovascular age-related macular degeneration
NMA	network meta-analysis
OR	odds ratio
PICOS	population, intervention, comparison, outcome measures, and study design
PRN	pro re nata
Q4W	every four weeks
Q8W	every eight weeks
Q12W	every twelve weeks
Q16W	every sixteen weeks
RAN	ranibizumab
RCT	randomized controlled trials
SD	standard deviation
SLR	systematic literature review
SUCRA	surface under the cumulative
TREX	treat and extend
UK	United Kingdom
