# Supplementary material for: Network Meta-Analysis of Bevacizumab Gamma Versus Competing Interventions for Treating Neovascular Age-Related Macular Degeneration in the United Kingdom
Source: J Mark Access Health Policy. 2025 Nov 19;13(4):58. doi: 10.3390/jmahp13040058 (PMC12641919; doi:10.3390/jmahp13040058)
Supplement: Supplementary file 1 [file jmahp-13-00058-s001.zip › jmahp-3716551-supplementary 17SEP2025_clean.pdf]

Supplementary Data

Table S1. Search strategy – original search

#	Query	Results from 25 Oct 2022
**Embase search strategy**		
1	Wet Macular Degeneration/	3,474
2	Choroidal Neovascularization/	5,596
3	age related macular degeneration/	13,595
4	(atrophy adj3 (choroidal or sclerosis or macul*)).tw.	2,681
5	((macul* or retina* or choroid* or neovascular*) adj4 degener*).tw.	48,029
6	(choroiditis adj2 tays).tw.	1
7	or/1-6	56,080
8	exp angiogenesis inhibitors/	349,333
9	exp angiogenesis inducing agents/	32,368
10	exp endothelial growth factors/	2,234
11	exp vascular endothelial growth factors/	153,899
12	(anti* adj3 VEGF*).tw.	18,736
13	(endothelial adj2 growth adj2 factor*).tw.	88,255
14	(Aflibercept$ or Eylea or Zaltrap or ALT L9 or ALT-L9 or ALTL9 or FYB203 or MYL 1710P or MYL-1710P or MYL1710P or M710 or CHS 2020 or CHS-2020 or CHS2020 or ABP 938 or ABP-938 or ABP938 or SB 15 or SB-15 or SB15 or SOK583A19 or CT P42 or CT-P42 or CTP42 or OT 702 or OT-702 or OT702).mp.	8,544
15	(Conbercept$ or lumitin).mp.	510
16	(Bevacizumab$ or Avastin or Zirabev or ABP215 or ABP 215 or ABP-215 or Cizumab or Bevacirel or "BCD 021" or BCD021 or BCD-021 or mAbxience or Krabeva or Zybev or Abevmy or Bevatas or Lytenava or ONS-5010 or ONS 5010 or ONS5010).mp.	71,470
17	(Ranibizumab$ or Ongavia or Lucentis or Razumab or Ranivisio or Ximluci or FYB201 or FYB 201 or FYB-201or SB11 or SB 11 or SB-11 or PF582 or PF 582 or PF-582 or LUBT010 or LUBT-010 or "LUBT 010" or Xlucane or BYOOVIZ).mp.	12,201
18	(Brolucizumab$ or Beovu or Vsiqq).mp.	441
19	(Pegaptanib$ or Macugen).mp.	2,501
20	(Faricimab$ or vabysmo).mp.	168
21	biosimilar$.mp.	10,947
22	or/8-21	522,161
23	Clinical trial/	1,047,631
24	exp Randomized controlled trial/	735,061
25	Randomization/	95,361
26	Prospective study/	804,137
27	Single blind procedure/	48,019
28	Double blind procedure/	199,982
29	Crossover procedure/	71,825
30	Placebo/	386,931
31	Controlled clinical trial/	467,512
32	(allocated adj2 random).ti,ab.	931
33	Placebo$.ti,ab.	350,424
34	(clinic$ adj trial$1).ti,ab.	646,256

#	Query	Results from 25 Oct 2022
35	((singl$ or doubl$ or treb$ or tripl$) adj (blind$3 or mask$3)).ti,ab.	269,158
36	rct.ti,ab.	49,147
37	Randomi?ed controlled trial$.ti,ab.	298,311
38	(random* or factorial* or crossover* or (cross adj over*) or placebo* or assign* or allocat* or volunteer*).ti,ab.	2,620,686
39	((singl* or doubl*) adj2 blind*).ti,ab.	263,899
40	(crossover procedure or double-blind procedure or randomized controlled trial or single-blind procedure).ti,ab.	126,888
41	or/23-40	4,348,080
42	7 and 22 and 41	3,512
43	exp animals/	29,258,744
44	(rat or rats or mouse or mice or murine).ti. or (nonhuman or in vitro study or in vivo study).hw.	8,402,923
45	or/43-44	31,508,310
46	exp human/	24,238,197
47	45 not 46	7,270,113
48	(letter or editorial or comment$ or note or case report).pt.	2,894,994
49	case study/ or letter/ or editorial/ or case report/	4,510,362
50	or/47-49	12,592,182
51	42 not 50	3,153
52	remove duplicates from 51	3,115
53	conference abstract.pt.	4,571,584
54	limit 53 to yr="1974 - 2019"	3,825,744
55	52 not 54	2,428
56	systematic review.pt. or systematic review.ti.	228,715
57	review.pt.	2,966,321
58	57 not 56	2,852,864
59	55 not 58	1,840
**Medline search strategy**		
1	Wet Macular Degeneration/	2,683
2	Choroidal Neovascularization/	6,600
3	(atrophy adj3 (choroidal or sclerosis or macul*)).tw.	1,839
4	((macul* or retina* or choroid* or neovascular*) adj4 degener*).tw.	35,832
5	(choroiditis adj2 tays).tw.	3
6	or/1-5	40,707
7	exp angiogenesis inhibitors/	65,504
8	exp angiogenesis inducing agents/	7,125
9	exp endothelial growth factors/	8,181
10	exp vascular endothelial growth factors/	62,356
11	(anti* adj3 VEGF*).tw.	11,365
12	(endothelial adj2 growth adj2 factor*).tw.	69,617
13	(Aflibercept$ or Eylea or Zaltrap or ALT L9 or ALT-L9 or ALTL9 or FYB203 or MYL 1710P or MYL-1710P or MYL1710P or M710 or CHS 2020 or CHS-2020 or CHS2020 or ABP 938 or ABP-938 or ABP938 or SB 15 or SB-15 or SB15 or SOK583A19 or CT P42 or CT-P42 or CTP42 or OT 702 or OT-702 or OT702).mp.	3,183
14	(Conbercept$ or lumitin).mp.	258
15	(Bevacizumab$ or Avastin or Zirabev or ABP215 or ABP 215 or ABP-215 or Cizumab or Bevacirel or "BCD 021" or BCD021 or BCD-021 or mAbxience or Krabeva or Zybev or	21,839

#	Query	Results from 25 Oct 2022
	Abevmy or Bevatas or Lytenava or ONS-5010 or ONS 5010 or ONS5010).mp.	
16	(Ranibizumab$ or Ongavia or Lucentis or Razumab or Ranivisio or Ximluci or FYB201 or FYB 201 or FYB-201or SB11 or SB 11 or SB-11 or PF582 or PF 582 or PF-582 or LUBT010 or LUBT-010 or "LUBT 010" or Xlucane or BYOOVIZ).mp.	6,124
17	(Brolucizumab$ or Beovu or Vsiqq).mp.	229
18	(Pegaptanib$ or Macugen).mp.	689
19	(Faricimab$ or vabysmo).mp.	51
20	biosimilar$.mp.	5,065
21	or/8-20	119,663
22	Clinical trial/	536,307
23	exp Randomized controlled trial/	580,962
24	Random allocation/ use medall	106,889
25	Double blind method/	173,391
26	Single blind method/	32,254
27	Placebos/	35,924
28	(allocated adj2 random).ti,ab.	823
29	Placebo$.ti,ab.	240,206
30	(clinic$ adj trial$1).ti,ab.	451,617
31	((singl$ or doubl$ or treb$ or tripl$) adj (blind$3 or mask$3)).ti,ab.	192,176
32	rct.ti,ab.	28,984
33	Randomi?ed controlled trial$.ti,ab.	231,284
34	(random* or factorial* or crossover* or (cross adj over*) or placebo* or assign* or allocat* or volunteer*).ti,ab.	1,961,354
35	((singl* or doubl*) adj2 blind*).ti,ab.	187,535
36	(crossover procedure or double-blind procedure or randomized controlled trial or single-blind procedure).ti,ab.	98,520
37	or/22-36	2,650,231
38	6 and 21 and 37	1,515
39	exp animals/	25,890,701
40	(rat or rats or mouse or mice or murine).ti. or (nonhuman or in vitro study or in vivo study).hw.	1,492,028
41	or/39-40	25,975,519
42	exp human/	20,831,921
43	41 not 42	5,143,598
44	(letter or editorial or comment$ or note or case report).pt.	2,104,656
45	case study/ or letter/ or editorial/ or case report/	3,893,892
46	or/43-45	9,210,694
47	38 not 46	1,428
48	remove duplicates from 47	1,426
49	systematic review.pt. or systematic review.ti.	256,363
50	review.pt.	3,063,226
51	50 not 49	2,929,337
52	48 not 51	1,120
**Cochrane CENTRAL search strategy**		
1	Macular Degeneration/	1,265
2	Choroidal Neovascularization/	448
3	age related macular degeneration/	1,265
4	(AMD or age related macular degeneration or wet macular degeneration or choroidal	4,079

#	Query	Results from 25 Oct 2022
	neovascularization).tw.	
5	or/1-4	4,289
6	(anti* adj3 VEGF*).tw.	1,742
7	(endothelial adj2 growth adj2 factor*).tw.	3,902
8	(Aflibercept$ or Eylea or Zaltrap or ALT L9 or ALT-L9 or ALTL9 or FYB203 or MYL 1710P or MYL-1710P or MYL1710P or M710 or CHS 2020 or CHS-2020 or CHS2020 or ABP 938 or ABP-938 or ABP938 or SB 15 or SB-15 or SB15 or SOK583A19 or CT P42 or CT-P42 or CTP42 or OT 702 or OT-702 or OT702).mp.	1,102
9	(Conbercept$ or lumitin).mp.	125
10	(Bevacizumab$ or Avastin or Zirabev or ABP215 or ABP 215 or ABP-215 or Cizumab or Bevacirel or "BCD 021" or BCD021 or BCD-021 or mAbxience or Krabeva or Zybev or Abevmy or Bevatas or Lytenava or ONS-5010 or ONS 5010 or ONS5010).mp.	7,446
11	(Ranibizumab$ or Ongavia or Lucentis or Razumab or Ranivisio or Ximluci or FYB201 or FYB 201 or FYB-201or SB11 or SB 11 or SB-11 or PF582 or PF 582 or PF-582 or LUBT010 or LUBT-010 or "LUBT 010" or Xlucane or BYOOVIZ).mp.	2,292
12	(Brolucizumab$ or Beovu or Vsiqq).mp.	77
13	(Pegaptanib$ or Macugen).mp.	188
14	(Faricimab$ or vabysmo).mp.	36
15	biosimilar$.mp.	1,572
16	or/6-15	14,622
17	5 and 16	1,779
18	random$.mp.	1,286,711
19	17 and 18	1,250
20	clinical trial protocol.pt.	28,827
21	19 not 20	1,240
**Cochrane CDSR search strategy**		
1	((macul* or retina* or choroid*) adj4 degener*).tw.	112
2	((macul* or retina* or choroid*) adj4 neovasc*).tw.	55
3	(atrophy adj3 (choroidal or sclerosis or macul*)).tw.	10
4	(AMD or age related macular degeneration or wet macular degeneration or choroidal neovascularization).tw.	112
5	or/1-4	143
6	(anti* adj3 VEGF*).tw.	66
7	(endothelial adj2 growth adj2 factor*).tw.	165
8	(Aflibercept$ or Eylea or Zaltrap or ALT L9 or ALT-L9 or ALTL9 or FYB203 or MYL 1710P or MYL-1710P or MYL1710P or M710 or CHS 2020 or CHS-2020 or CHS2020 or ABP 938 or ABP-938 or ABP938 or SB 15 or SB-15 or SB15 or SOK583A19 or CT P42 or CT-P42 or CTP42 or OT 702 or OT-702 or OT702).mp.	29
9	(Conbercept$ or lumitin).mp.	7
10	(Bevacizumab$ or Avastin or Zirabev or ABP215 or ABP 215 or ABP-215 or Cizumab or Bevacirel or "BCD 021" or BCD021 or BCD-021 or mAbxience or Krabeva or Zybev or Abevmy or Bevatas or Lytenava or ONS-5010 or ONS 5010 or ONS5010).mp.	112
11	(Ranibizumab$ or Ongavia or Lucentis or Razumab or Ranivisio or Ximluci or FYB201 or FYB 201 or FYB-201or SB11 or SB 11 or SB-11 or PF582 or PF 582 or PF-582 or LUBT010 or LUBT-010 or "LUBT 010" or Xlucane or BYOOVIZ).mp.	34
12	(Brolucizumab$ or Beovu or Vsiqq).mp.	6
13	(Pegaptanib$ or Macugen).mp.	21
14	(Faricimab$ or vabysmo).mp.	0

#	Query	Results from 25 Oct 2022
15	biosimilar$.mp.	28
16	or/6-15	242
17	5 and 16	57

Table S2: Search strategy – updated search

#	Query	Results from 30 Jan 2024
**Embase search strategy**		
1	Wet Macular Degeneration/	4,129
2	Choroidal Neovascularization/	6,356
3	age related macular degeneration/	16,018
4	((choroidal or sclerosis or macul*) adj3 atrophy).tw.	2,965
5	((macul* or retina* or choroid* or neovascular*) adj4 (neovascular* or degener*)).tw.	93,157
6	((wet or neovascul* or exudative) adj3 AMD).tw.	5,957
7	(choroiditis adj2 tays).tw.	1
8	or/1-7	99,769
9	exp angiogenesis inhibitors/	556,244
10	exp angiogenesis inducing agents/	40,830
11	exp endothelial growth factors/	2,272
12	exp vascular endothelial growth factors/	167,725
13	(anti* adj3 VEGF*).tw.	20,593
14	(endothelial adj2 growth adj2 factor*).tw.	93,891
15	(Aflibercept$ or Eylea or Zaltrap or ALT L9 or ALT-L9 or ALTL9 or FYB203 or MYL 1710P or MYL-1710P or MYL1710P or M710 or CHS 2020 or CHS-2020 or CHS2020 or ABP 938 or ABP-938 or ABP938 or SB 15 or SB-15 or SB15 or SOK583A19 or CT P42 or CT-P42 or CTP42 or OT 702 or OT-702 or OT702).mp.	9,876
16	(Conbercept$ or lumitin).mp.	702
17	(Bevacizumab$ or Avastin or Zirabev or ABP215 or ABP 215 or ABP-215 or Cizumab or Bevacirel or "BCD 021" or BCD021 or BCD-021 or mAbxience or Krabeva or Zybev or Abevmy or Bevatas or Lytenava or ONS-5010 or ONS 5010 or ONS5010).mp.	79,099
18	(Ranibizumab$ or Ongavia or Lucentis or Razumab or Ranivisio or Ximluci or FYB201 or FYB 201 or FYB-201or SB11 or SB 11 or SB-11 or PF582 or PF 582 or PF-582 or LUBT010 or LUBT-010 or "LUBT 010" or Xlucane or BYOOVIZ).mp.	13,373
19	(Brolucizumab$ or Beovu or Vsiqq).mp.	672
20	(Pegaptanib$ or Macugen).mp.	2,630
21	(Faricimab$ or vabysmo).mp.	371
22	biosimilar$.mp.	12,521
23	or/9-22	747,672
24	Clinical trial/	1,078,717
25	exp Randomized controlled trial/	807,486
26	Randomization/	99,099
27	Prospective study/	904,176
28	Single blind procedure/	53,359
29	Double blind procedure/	215,449
30	Crossover procedure/	76,755
31	Placebo/	408,418

#	Query	Results from 30 Jan 2024
32	Controlled clinical trial/	472,168
33	(allocated adj2 random).ti,ab.	959
34	Placebo$.ti,ab.	374,375
35	(clinic$ adj trial$1).ti,ab.	718,679
36	((singl$ or doubl$ or treb$ or tripl$) adj (blind$3 or mask$3)).ti,ab.	286,654
37	rct.ti,ab.	56,142
38	Randomi?ed controlled trial$.ti,ab.	337,021
39	(random* or factorial* or crossover* or (cross adj over*) or placebo* or assign* or allocate* or volunteer*).ti,ab.	2,744,336
40	((singl* or doubl*) adj2 blind*).ti,ab.	280,835
41	(crossover procedure or double-blind procedure or randomized controlled trial or single-blind procedure).ti,ab.	143,271
42	or/24-41	4,620,842
43	8 and 23 and 42	6,016
44	exp animals/	31,322,869
45	(rat or rats or mouse or mice or murine).ti. or (nonhuman or in vitro study or in vivo study).hw.	8,978,553
46	or/44-45	33,730,540
47	exp human/	26,117,180
48	46 not 47	7,613,360
49	(letter or editorial or comment$ or note or case report).pt.	3,076,169
50	case study/ or letter/ or editorial/ or case report/	4,786,071
51	or/48-50	13,267,364
52	43 not 51	4,949
53	remove duplicates from 52	4,891
54	conference abstract.pt.	5,034,529
55	limit 54 to yr="1974 - 2019"	3,859,597
56	53 not 55	3,888
57	systematic review.pt. or systematic review.ti. or meta-analys$s.pt. or meta-analys$s.ti. or (meta adj2 analys$s).ti.	276,833
58	review.pt.	3,179,234
59	58 not 57	3,039,772
60	56 not 59	2,893
**Medline search strategy**		
1	Wet Macular Degeneration/	2,955
2	Choroidal Neovascularization/	6,909
3	((choroidal or sclerosis or macul*) adj3 atrophy).tw.	2,026
4	((macul* or retina* or choroid* or neovascular*) adj4 (neovascular* or degener*)).tw.	69,362
5	((wet or neovascul* or exudative) adj3 AMD).tw.	4,033
6	(choroiditis adj2 tays).tw.	3
7	or/1-6	71,692
8	exp angiogenesis inhibitors/	68,487
9	exp angiogenesis inducing agents/	7,287
10	exp endothelial growth factors/	8,320
11	exp vascular endothelial growth factors/	65,815
12	(anti* adj3 VEGF*).tw.	12,552
13	(endothelial adj2 growth adj2 factor*).tw.	73,635
14	(Aflibercept$ or Eylea or Zaltrap or ALT L9 or ALT-L9 or ALTL9 or FYB203 or MYL	3,653

#	Query	Results from 30 Jan 2024
	1710P or MYL-1710P or MYL1710P or M710 or CHS 2020 or CHS-2020 or CHS2020 or ABP 938 or ABP-938 or ABP938 or SB 15 or SB-15 or SB15 or SOK583A19 or CT P42 or CT-P42 or CTP42 or OT 702 or OT-702 or OT702).mp.	
15	(Conbercept$ or lumitin).mp.	329
16	(Bevacizumab$ or Avastin or Zirabev or ABP215 or ABP 215 or ABP-215 or Cizumab or Bevacirel or "BCD 021" or BCD021 or BCD-021 or mAbxience or Krabeva or Zybev or Abevmy or Bevatas or Lytenava or ONS-5010 or ONS 5010 or ONS5010).mp.	23,754
17	(Ranibizumab$ or Ongavia or Lucentis or Razumab or Ranivisio or Ximluci or FYB201 or FYB 201 or FYB-201or SB11 or SB 11 or SB-11 or PF582 or PF 582 or PF-582 or LUBT010 or LUBT-010 or "LUBT 010" or Xlucane or BYOOVIZ).mp.	6,616
18	(Brolucizumab$ or Beovu or Vsiqq).mp.	360
19	(Pegaptanib$ or Macugen).mp.	701
20	(Faricimab$ or vabysmo).mp.	126
21	biosimilar$.mp.	5,799
22	or/8-21	168,835
23	Clinical trial/	539,424
24	exp Randomized controlled trial/	609,549
25	Random allocation/	107,059
26	Double blind method/	177,396
27	Single blind method/	33,226
28	Placebos/	35,934
29	(allocated adj2 random).ti,ab.	842
30	Placebo$.ti,ab.	253,288
31	(clinic$ adj trial$1).ti,ab.	500,020
32	((singl$ or doubl$ or treb$ or tripl$) adj (blind$3 or mask$3)).ti,ab.	202,606
33	rct.ti,ab.	33,336
34	Randomi?ed controlled trial$.ti,ab.	261,947
35	(random* or factorial* or crossover* or (cross adj over*) or placebo* or assign* or allocat* or volunteer*).ti,ab.	2,125,812
36	((singl* or doubl*) adj2 blind*).ti,ab.	197,532
37	(crossover procedure or double-blind procedure or randomized controlled trial or single-blind procedure).ti,ab.	111,576
38	or/23-37	2,844,764
39	7 and 22 and 38	2,576
40	exp animals/	26,943,596
41	(rat or rats or mouse or mice or murine).ti. or (nonhuman or in vitro study or in vivo study).hw.	1,537,689
42	or/40-41	27,040,307
43	exp human/	21,751,033
44	42 not 43	5,289,274
45	(letter or editorial or comment$ or note or case report).pt.	2,223,108
46	case study/ or letter/ or editorial/ or case report/	4,076,224
47	or/44-46	9,552,720
48	39 not 47	2,170
49	remove duplicates from 48	2,163
50	systematic review.pt. or systematic review.ti. or meta-analys$s.pt. or meta-analys$s.ti. or (meta adj2 analys$s).ti.	307,963
51	review.pt.	3,272,733

#	Query	Results from 30 Jan 2024
52	51 not 50	3,113,098
53	49 not 52	1,657
**Cochrane CENTRAL search strategy**		
1	Wet Macular Degeneration/	516
2	Choroidal Neovascularization/	494
3	((choroidal or sclerosis or macul*) adj3 atrophy).tw.	126
4	((macul* or retina* or choroid* or neovascular*) adj4 (neovascular* or degener*)).tw.	5,080
5	((wet or neovascul* or exudative) adj3 AMD).tw.	894
6	(choroiditis adj2 tays).tw.	0
7	or/1-6	5,227
8	(anti* adj3 VEGF*).tw.	1,800
9	(endothelial adj2 growth adj2 factor*).tw.	3,963
10	(Aflibercept$ or Eylea or Zaltrap or ALT L9 or ALT-L9 or ALTL9 or FYB203 or MYL 1710P or MYL-1710P or MYL1710P or M710 or CHS 2020 or CHS-2020 or CHS2020 or ABP 938 or ABP-938 or ABP938 or SB 15 or SB-15 or SB15 or SOK583A19 or CT P42 or CT-P42 or CTP42 or OT 702 or OT-702 or OT702).mp.	1,185
11	(Conbercept$ or lumitin).mp.	159
12	(Bevacizumab$ or Avastin or Zirabev or ABP215 or ABP 215 or ABP-215 or Cizumab or Bevacirel or "BCD 021" or BCD021 or BCD-021 or mAbxience or Krabeva or Zybev or Abevmy or Bevatas or Lytenava or ONS-5010 or ONS 5010 or ONS5010).mp.	7,617
13	(Ranibizumab$ or Ongavia or Lucentis or Razumab or Ranivisio or Ximluci or FYB201 or FYB 201 or FYB-201or SB11 or SB 11 or SB-11 or PF582 or PF 582 or PF-582 or LUBT010 or LUBT-010 or "LUBT 010" or Xlucane or BYOOVIZ).mp.	2,320
14	(Brolucizumab$ or Beovu or Vsiqq).mp.	90
15	(Pegaptanib$ or Macugen).mp.	174
16	(Faricimab$ or vabysmo).mp.	68
17	biosimilar$.mp.	1,657
18	or/8-17	15,100
19	7 and 18	2,237
20	random$.mp.	1,356,398
21	19 and 20	1,583
22	clinical trial protocol.pt.	577,426
23	21 not 22	993
**Cochrane CDSR search strategy**		
1	((choroidal or sclerosis or macul*) adj3 atrophy).tw.	12
2	((macul* or retina* or choroid* or neovascular*) adj4 (neovascular* or degener*)).tw.	203
3	((wet or neovascul* or exudative) adj3 AMD).tw.	30
4	(choroiditis adj2 tays).tw.	0
5	or/1-4	205
6	(anti* adj3 VEGF*).tw.	72
7	(endothelial adj2 growth adj2 factor*).tw.	173
8	(Aflibercept$ or Eylea or Zaltrap or ALT L9 or ALT-L9 or ALTL9 or FYB203 or MYL 1710P or MYL-1710P or MYL1710P or M710 or CHS 2020 or CHS-2020 or CHS2020 or ABP 938 or ABP-938 or ABP938 or SB 15 or SB-15 or SB15 or SOK583A19 or CT P42 or CT-P42 or CTP42 or OT 702 or OT-702 or OT702).mp.	31
9	(Conbercept$ or lumitin).mp.	10
10	(Bevacizumab$ or Avastin or Zirabev or ABP215 or ABP 215 or ABP-215 or Cizumab or Bevacirel or "BCD 021" or BCD021 or BCD-021 or mAbxience or Krabeva or Zybev or	118

#	**Query**	**Results from 30 Jan 2024**
	Abevmy or Bevatas or Lytenava or ONS-5010 or ONS 5010 or ONS5010).mp.	
11	(Ranibizumab$ or Ongavia or Lucentis or Razumab or Ranivisio or Ximluci or FYB201 or FYB 201 or FYB-201or SB11 or SB 11 or SB-11 or PF582 or PF 582 or PF-582 or LUBT010 or LUBT-010 or "LUBT 010" or Xlucane or BYOOVIZ).mp.	36
12	(Brolucizumab$ or Beovu or Vsiqq).mp.	11
13	(Pegaptanib$ or Macugen).mp.	21
14	(Faricimab$ or vabysmo).mp.	6
15	biosimilar$.mp.	33
16	or/6-15	258
17	5 and 16	75

Figure S1. RoB assessment results

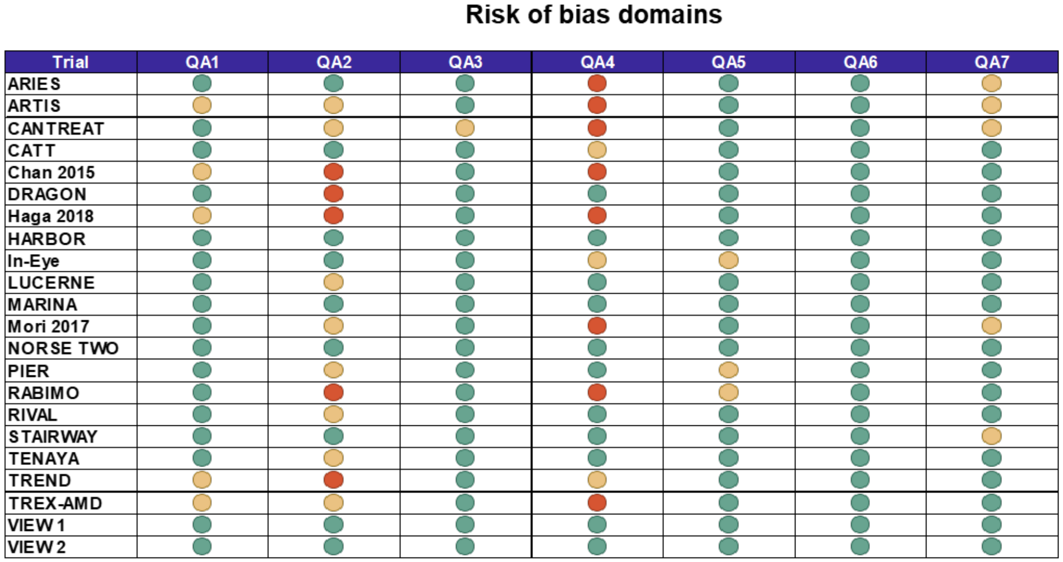

(A) Risk of bias summary: review authors' judgments about each risk of bias item for each included study.
(B) Risk of bias graph: review authors' judgments about each risk of bias item presented as percentages across all included studies.

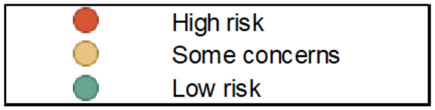

A

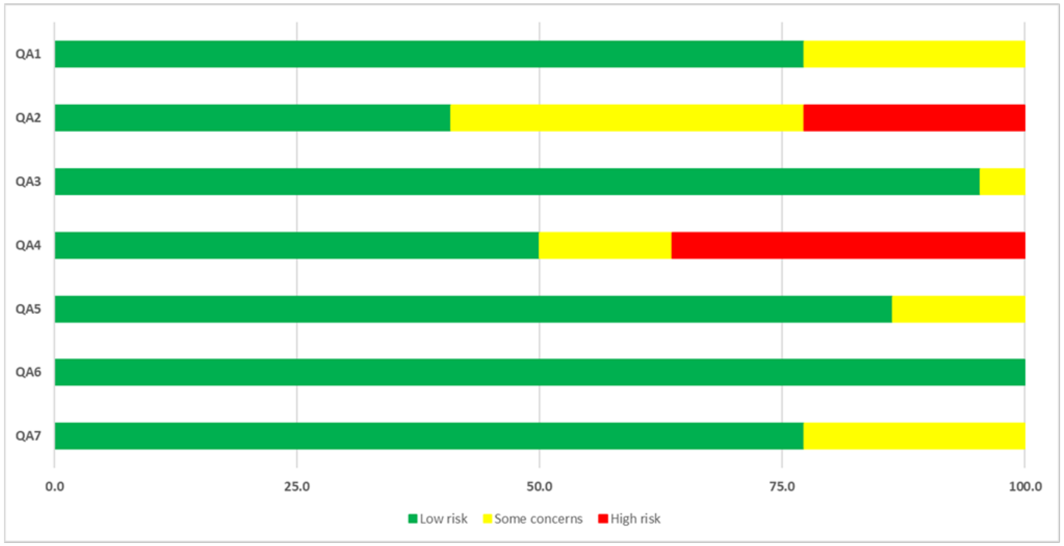

Domains:
QA1: Adequacy of random allocation method.
QA2: Adequacy of allocation concealment.
QA3: Similarity of groups at baseline.
QA4: Blinding of care providers, participants, and assessors.
QA5: Imbalances in dropouts and their explanation/adjustment.
QA6: Evidence of selective outcome reporting.
QA7: Use and appropriateness of ITT analysis

B

Table S3. Trial-level input data contributing to the network meta-analysis of change from baseline in BCVA at 12 months

Study	Treatment	Total N	Change from baseline in BCVA	Change from baseline SD	Change from baseline SE
ARIES	AFLI 2.0 TREX	106	**7.800**	**13.930**	1.350
ARIES	AFLI 2.0 Q8W	104	**10.200**	**13.930**	1.370
ARTIS	RAN 0.5 PRN	45	7.800	16.000	2.390
ARTIS	RAN 0.5 PRN load	49	10.900	15.800	2.260
CANTREAT	RAN 0.5 TREX	268	8.400	11.900	0.730
CANTREAT	RAN 0.5 Q4W	258	6.000	11.900	0.740
CATT	RAN 0.5 Q4W	284	8.500	14.100	0.800
CATT	RAN 0.5 PRN	285	6.800	13.100	0.800
DRAGON	RAN 0.5 Q4W	167	12.300	**13.930**	0.956
DRAGON	RAN 0.5 PRN load	166	9.600	**13.930**	1.038
HARBOR	RAN 0.5 Q4W	275	10.100	13.300	0.800
HARBOR	RAN 0.5 PRN load	275	8.200	13.300	0.800
Haga 2018	AFLI 2.0 TREX	21	15.900	**13.930**	3.040
Haga 2018	AFLI 2.0 Q8W	20	13.100	**13.930**	3.110
In-Eye	RAN 0.5 Q8W	103	7.000	**13.930**	1.370
In-Eye	RAN 0.5 TREX	99	6.700	**13.930**	1.400
In-Eye	RAN 0.5 PRN load	104	7.300	**13.930**	1.370
LUCERNE	FAR 6.0 Q8-Q16W	331	6.600	**13.930**	0.770
LUCERNE	AFLI 2.0 Q8W	327	6.600	**13.930**	0.770
MARINA	RAN 0.5 Q4W	240	7.200	**13.930**	0.900
MARINA	SHAM	238	-10.400	**13.930**	0.900
Mori 2017	AFLI 2.0 PRN	30	3.400	**13.930**	2.540
Mori 2017	AFLI 2.0 Q8W	28	7.100	**13.930**	2.630
NORSE TWO	BEV-g 1.25 Q4W	104	11.200	12.190	1.200
NORSE TWO	RAN 0.5 Q12W	96	5.800	14.800	1.510
PIER	SHAM	63	-16.300	22.300	2.810
PIER	RAN 0.5 Q12W	61	-0.200	13.100	1.680
RABIMO	RAN 0.5 Q8W	20	8.500	**13.930**	3.110
RABIMO	RAN 0.5 PRN loading	20	6.500	**13.930**	3.110
RIVAL	RAN 0.5 TREX	127	6.900	12.250	1.090
RIVAL	AFLI 2.0 TREX	121	5.200	12.830	1.170
STAIRWAY	RAN 0.5 Q4W	16	9.600	**13.930**	3.480
STAIRWAY	FAR 6.0 Q12W	24	10.100	**13.930**	2.840
STAIRWAY	FAR 6.0 Q16W	31	11.400	**13.930**	2.500
TENAYA	FAR 6.0 Q8-Q16W	334	5.800	**13.930**	0.760
TENAYA	AFLI 2.0 Q8W	337	5.100	**13.930**	0.760
TREND	RAN 0.5 TREX	294	6.600	**13.930**	0.810
TREND	RAN 0.5 Q4W	295	7.900	**13.930**	0.810
TREX-AMD	RAN 0.5 Q4W	20	9.200	**13.930**	3.110
TREX-AMD	RAN 0.5 TREX	37	9.900	**13.930**	2.290
VIEW 1	RAN 0.5 Q4W	304	8.100	15.300	0.880
VIEW 1	AFLI 2.0 Q8W	301	7.900	15.000	0.860
VIEW 2	RAN 0.5 Q4W	291	9.400	13.500	0.790

VIEW 2	AFLI 2.0 Q8W	306	8.900	14.400	0.820

Note: Imputed SDs and calculated CFB values are displayed in bold

Table S4. Change from baseline in BCVA at 12 months; mean differences (95% CrI) for all possible pairs of interventions

RAN 0.5 Q4W	-3.42 (-10.66, 3.84)	-0.27 (-2.35, 1.80)	-1.55 (-4.59, 1.53)	3.84 (-3.73, 11.46)	0.09 (-2.92, 3.03)	0.24 (-7.78, 8.19)	1.46 (-6.21, 9.15)	-2.08 (-4.98, 0.68)	-1.43 (-3.38, 0.66)	-0.42 (-4.12, 3.40)	-1.35 (-7.98, 5.27)	0.24 (-1.62, 2.00)	**-17.21 (-20.33, -13.86)**
3.42 (-3.84, 10.66)	AFLI 2.0 PRN	3.16 (-3.87, 10.16)	1.88 (-5.72, 9.50)	7.28 (-3.26, 17.80)	3.51 (-3.84, 10.87)	3.68 (-7.13, 14.41)	4.90 (-5.66, 15.45)	1.33 (-6.46, 9.06)	2.01 (-5.49, 9.51)	3.03 (-5.10, 11.15)	2.07 (-7.77, 11.93)	3.66 (-3.77, 11.04)	**-13.76 (-21.63, -5.81)**
0.27 (-1.80, 2.35)	-3.16 (-10.16, 3.87)	AFLI 2.0 Q8W	-1.27 (-4.26, 1.76)	4.11 (-3.76, 12.05)	0.36 (-1.82, 2.53)	0.51 (-7.78, 8.74)	1.73 (-6.19, 9.70)	-1.81 (-5.40, 1.62)	-1.16 (-3.94, 1.78)	-0.14 (-4.33, 4.12)	-1.09 (-8.02, 5.87)	0.50 (-2.11, 3.06)	**-16.95 (-20.66, -12.95)**
1.55 (-1.53, 4.59)	-1.88 (-9.50, 5.72)	1.27 (-1.76, 4.26)	AFLI 2.0 TREX	5.38 (-2.86, 13.62)	1.64 (-2.11, 5.30)	1.79 (-6.83, 10.30)	3.01 (-5.30, 11.26)	-0.54 (-4.80, 3.55)	0.12 (-3.40, 3.70)	1.13 (-3.47, 5.79)	0.17 (-7.11, 7.52)	1.77 (-1.28, 4.72)	**-15.66 (-20.04, -11.15)**
-3.84 (-11.46, 3.73)	-7.28 (-17.80, 3.26)	-4.11 (-12.05, 3.76)	-5.38 (-13.62, 2.86)	BEV g 1.25 Q4W	-3.76 (-11.97, 4.41)	-3.62 (-14.58, 7.42)	-2.39 (-13.14, 8.43)	-5.93 (-14.07, 2.10)	-5.26 (-13.10, 2.61)	-4.28 (-12.73, 4.19)	**-5.23 (-9.47, -0.91)**	-3.61 (-11.47, 4.17)	**-21.05 (-28.25, -13.73)**
-0.09 (-3.03, 2.92)	-3.51 (-10.87, 3.84)	-0.36 (-2.53, 1.82)	-1.64 (-5.30, 2.11)	3.76 (-4.41, 11.97)	FAR 6.0 Q8-Q16W	0.15 (-8.40, 8.63)	1.37 (-6.86, 9.61)	-2.16 (-6.33, 1.85)	-1.53 (-4.99, 2.17)	-0.51 (-5.20, 4.28)	-1.45 (-8.69, 5.85)	0.14 (-3.22, 3.48)	**-17.30 (-21.59, -12.74)**
-0.24 (-8.19, 7.78)	-3.68 (-14.41, 7.13)	-0.51 (-8.74, 7.78)	-1.79 (-10.30, 6.83)	3.62 (-7.42, 14.58)	-0.15 (-8.63, 8.40)	FAR 6.0 Q12W	1.24 (-6.25, 8.65)	-2.33 (-10.82, 6.12)	-1.65 (-9.85, 6.61)	-0.65 (-9.44, 8.19)	-1.58 (-12.00, 8.80)	-0.01 (-8.19, 8.15)	**-17.42 (-25.99, -8.77)**
-1.46 (-9.15, 6.21)	-4.90 (-15.45, 5.66)	-1.73 (-9.70, 6.19)	-3.01 (-11.26, 5.30)	2.39 (-8.43, 13.14)	-1.37 (-9.61, 6.86)	-1.24 (-8.65, 6.25)	FAR 6.0 Q16W	-3.56 (-11.78, 4.59)	-2.87 (-10.82, 5.08)	-1.87 (-10.39, 6.65)	-2.84 (-12.97, 7.30)	-1.24 (-9.13, 6.63)	**-18.67 (-26.94, -10.26)**
2.08 (-0.68, 4.98)	-1.33 (-9.06, 6.46)	1.81 (-1.62, 5.40)	0.54 (-3.55, 4.80)	5.93 (-2.10, 14.07)	2.16 (-1.85, 6.33)	2.33 (-6.12, 10.82)	3.56 (-4.59, 11.78)	RAN 0.5 PRN	0.66 (-2.48, 4.07)	1.67 (-2.83, 6.36)	0.72 (-6.42, 8.01)	2.32 (-0.99, 5.67)	**-15.13 (-19.24, -10.59)**
1.43 (-0.66, 3.38)	-2.01 (-9.51, 5.49)	1.16 (-1.78, 3.94)	-0.12 (-3.70, 3.40)	5.26 (-2.61, 13.10)	1.53 (-2.17, 4.99)	1.65 (-6.61, 9.85)	2.87 (-5.08, 10.82)	-0.66 (-4.07, 2.48)	RAN 0.5 PRN load	1.01 (-2.60, 4.58)	0.06 (-6.87, 6.96)	1.66 (-0.91, 4.02)	**-15.78 (-19.53, -11.96)**
0.42 (-3.40, 4.12)	-3.03 (-11.15, 5.10)	0.14 (-4.12, 4.33)	-1.13 (-5.79, 3.47)	4.28 (-4.19, 12.73)	0.51 (-4.28, 5.20)	0.65 (-8.19, 9.44)	1.87 (-6.65, 10.39)	-1.67 (-6.36, 2.83)	-1.01 (-4.58, 2.60)	RAN 0.5 Q8W	-0.95 (-8.56, 6.63)	0.65 (-3.18, 4.33)	**-16.79 (-21.64, -11.82)**
1.35 (-5.27, 7.98)	-2.07 (-11.93, 7.77)	1.09 (-5.87, 8.02)	-0.17 (-7.52, 7.11)	**5.23 (0.91, 9.47)**	1.45 (-5.85, 8.69)	1.58 (-8.80, 12.00)	2.84 (-7.30, 12.97)	-0.72 (-8.01, 6.42)	-0.06 (-6.96, 6.87)	0.95 (-6.63, 8.56)	RAN 0.5 Q12W	1.59 (-5.33, 8.40)	**-15.84 (-21.92, -9.63)**
-0.24 (-2.00, 1.62)	-3.66 (-11.04, 3.77)	-0.50 (-3.06, 2.11)	-1.77 (-4.72, 1.28)	3.61 (-4.17, 11.47)	-0.14 (-3.48, 3.22)	0.01 (-8.15, 8.19)	1.24 (-6.63, 9.13)	-2.32 (-5.67, 0.99)	-1.66 (-4.02, 0.91)	-0.65 (-4.33, 3.18)	-1.59 (-8.40, 5.33)	RAN 0.5 TREX	**-17.45 (-20.99, -13.56)**
**17.21 (13.86, 20.33)**	**13.76 (5.81, 21.63)**	**16.95 (12.95, 20.66)**	**15.66 (11.15, 20.04)**	**21.05 (13.73, 28.25)**	**17.30 (12.74, 21.59)**	**17.42 (8.77, 25.99)**	**18.67 (10.26, 26.94)**	**15.13 (10.59, 19.24)**	**15.78 (11.96, 19.53)**	**16.79 (11.82, 21.64)**	**15.84 (9.63, 21.92)**	**17.45 (13.56, 20.99)**	SHAM

Key: AFLI – aflibercept; BCVA – best-corrected visual acuity; BEV – bevacizumab; CrI – credible interval; FAR – faricimab; PRN – *pro re nata*; RAN – ranibizumab; TREX – treat and extend.

Note: Values presented are mean differences and 95% CrI of the column intervention compared to the row intervention.

Table S5. Proportion of patients gaining at least 15 letters at 12 months; OR (95% CrI) for all possible pairs of interventions

RAN 0.5 Q4W	0.97 (0.67, 1.41)	0.77 (0.44, 1.37)	0.36 (0.07, 1.86)	1.04 (0.60, 1.84)	0.83 (0.19, 3.60)	1.48 (0.39, 5.82)	**0.59 (0.35, 0.95)**	0.89 (0.64, 1.30)	0.73 (0.38, 1.40)	**0.15 (0.04, 0.64)**	1.00 (0.71, 1.39)	**0.10 (0.05, 0.22)**
1.03 (0.71, 1.49)	AFLI 2.0 Q8W	0.80 (0.47, 1.36)	0.37 (0.07, 1.99)	1.07 (0.70, 1.64)	0.86 (0.19, 3.86)	1.52 (0.38, 6.33)	0.61 (0.32, 1.10)	0.92 (0.57, 1.55)	0.75 (0.36, 1.57)	**0.15 (0.03, 0.70)**	1.03 (0.64, 1.63)	**0.11 (0.04, 0.24)**
1.29 (0.73, 2.27)	1.25 (0.73, 2.14)	AFLI 2.0 TREX	0.47 (0.08, 2.64)	1.35 (0.68, 2.67)	1.07 (0.22, 5.13)	1.91 (0.45, 8.45)	0.76 (0.36, 1.58)	1.15 (0.61, 2.24)	0.95 (0.41, 2.16)	**0.19 (0.04, 0.93)**	1.29 (0.73, 2.26)	**0.13 (0.05, 0.34)**
2.77 (0.54, 13.90)	2.69 (0.50, 13.97)	2.14 (0.38, 11.77)	BEV g 1.25 Q4W	2.88 (0.51, 15.91)	2.29 (0.26, 20.32)	4.09 (0.49, 34.13)	1.63 (0.29, 8.70)	2.47 (0.46, 13.05)	2.02 (0.35, 11.53)	**0.42 (0.20, 0.86)**	2.75 (0.51, 14.36)	0.28 (0.07, 1.15)
0.96 (0.54, 1.67)	0.93 (0.61, 1.42)	0.74 (0.37, 1.47)	0.35 (0.06, 1.95)	FAR 6.0 Q8-Q16W	0.80 (0.17, 3.81)	1.42 (0.33, 6.25)	0.57 (0.26, 1.17)	0.86 (0.45, 1.68)	0.70 (0.30, 1.64)	**0.14 (0.03, 0.69)**	0.96 (0.51, 1.78)	**0.10 (0.04, 0.25)**
1.21 (0.28, 5.23)	1.17 (0.26, 5.30)	0.93 (0.19, 4.47)	0.44 (0.05, 3.89)	1.25 (0.26, 6.03)	FAR 6.0 Q12W	1.77 (0.51, 6.55)	0.71 (0.15, 3.31)	1.08 (0.24, 4.88)	0.88 (0.18, 4.36)	0.18 (0.02, 1.42)	1.20 (0.27, 5.40)	**0.12 (0.02, 0.63)**
0.68 (0.17, 2.57)	0.66 (0.16, 2.62)	0.52 (0.12, 2.24)	0.24 (0.03, 2.04)	0.70 (0.16, 2.99)	0.56 (0.15, 1.97)	FAR 6.0 Q16W	0.40 (0.09, 1.65)	0.61 (0.15, 2.40)	0.49 (0.11, 2.17)	**0.10 (0.01, 0.73)**	0.67 (0.16, 2.68)	**0.07 (0.01, 0.32)**
**1.70 (1.05, 2.83)**	1.64 (0.91, 3.12)	1.31 (0.63, 2.82)	0.61 (0.11, 3.43)	1.76 (0.85, 3.83)	1.41 (0.30, 6.72)	2.51 (0.61, 10.92)	RAN 0.5 PRN	1.51 (0.90, 2.73)	1.24 (0.57, 2.77)	0.25 (0.06, 1.20)	1.69 (0.95, 3.09)	**0.17 (0.07, 0.43)**
1.12 (0.77, 1.56)	1.09 (0.64, 1.76)	0.87 (0.45, 1.63)	0.41 (0.08, 2.15)	1.17 (0.59, 2.20)	0.93 (0.20, 4.16)	1.65 (0.42, 6.80)	0.66 (0.37, 1.11)	RAN 0.5 PRN load	0.82 (0.43, 1.50)	**0.17 (0.04, 0.75)**	1.12 (0.71, 1.68)	**0.11 (0.05, 0.26)**
1.37 (0.71, 2.63)	1.33 (0.64, 2.77)	1.06 (0.46, 2.44)	0.50 (0.09, 2.88)	1.42 (0.61, 3.33)	1.14 (0.23, 5.62)	2.02 (0.46, 9.29)	0.81 (0.36, 1.76)	1.22 (0.67, 2.31)	RAN 0.5 Q8W	0.21 (0.04, 1.02)	1.36 (0.71, 2.60)	**0.14 (0.05, 0.38)**
**6.70 (1.55, 28.17)**	**6.49 (1.44, 28.87)**	**5.17 (1.08, 24.37)**	**2.41 (1.16, 5.09)**	**6.97 (1.46, 32.81)**	5.54 (0.71, 43.27)	**9.85 (1.37, 72.73)**	3.94 (0.84, 17.94)	**5.98 (1.34, 26.68)**	4.88 (0.98, 23.87)	RAN 0.5 Q12W	**6.65 (1.48, 29.47)**	0.68 (0.19, 2.28)
1.00 (0.72, 1.41)	0.97 (0.61, 1.57)	0.78 (0.44, 1.37)	0.36 (0.07, 1.95)	1.04 (0.56, 1.98)	0.83 (0.19, 3.74)	1.48 (0.37, 6.11)	0.59 (0.32, 1.05)	0.89 (0.60, 1.41)	0.73 (0.38, 1.40)	0.15 (0.03, 0.68)	RAN 0.5 TREX	**0.10 (0.04, 0.23)**
**9.79 (4.63, 21.89)**	**9.51 (4.12, 22.95)**	**7.59 (2.97, 20.49)**	3.55 (0.87, 15.36)	**10.20 (4.00, 27.31)**	**8.17 (1.58, 43.05)**	**14.59 (3.13, 71.52)**	**5.78 (2.33, 14.71)**	**8.73 (3.89, 21.38)**	**7.17 (2.65, 20.17)**	1.47 (0.44, 5.23)	**9.78 (4.28, 23.34)**	SHAM

Key: AFLI – aflibercept; BEV – bevacizumab; CrI – credible interval; FAR – faricimab; OR – odds ratio; PRN – *pro re nata*; RAN – ranibizumab; TREX – treat and extend.
Note: Values presented are ORs and 95% CrI of the column intervention compared to the row intervention.

Table S6. Proportion of patients losing fewer than 15 letters at 12 months; OR (95% CrI) for all possible pairs of interventions

RAN 0.5 Q4W	1.14 (0.44, 3.01)	0.44 (0.06, 2.95)	3.03 (0.29, 44.21)	1.11 (0.28, 4.28)	1.08 (0.32, 3.53)	0.72 (0.30, 1.83)	0.63 (0.14, 2.61)	0.96 (0.15, 8.41)	0.71 (0.27, 1.66)	**0.09 (0.03, 0.41)**
0.88 (0.33, 2.26)	AFLI 2.0 Q8W	0.38 (0.04, 3.23)	2.68 (0.21, 45.26)	0.97 (0.34, 2.58)	0.95 (0.20, 4.17)	0.63 (0.17, 2.39)	0.55 (0.09, 3.02)	0.84 (0.10, 8.56)	0.62 (0.15, 2.20)	**0.08 (0.02, 0.50)**
2.30 (0.34, 17.18)	2.63 (0.31, 24.70)	AFLI 2.0 TREX	6.99 (0.33, 226.67)	2.56 (0.24, 29.03)	2.50 (0.26, 24.61)	1.65 (0.22, 14.04)	1.44 (0.15, 13.42)	2.20 (0.15, 46.88)	1.62 (0.28, 9.57)	0.21 (0.02, 3.08)
0.33 (0.02, 3.48)	0.37 (0.02, 4.80)	0.14 (0.00, 3.02)	BEV g 1.25 Q4W	0.37 (0.02, 5.53)	0.35 (0.02, 4.84)	0.24 (0.01, 3.01)	0.21 (0.01, 3.17)	0.32 (0.07, 1.35)	0.23 (0.01, 2.81)	**0.03 (0.00, 0.24)**
0.90 (0.23, 3.51)	1.03 (0.39, 2.92)	0.39 (0.03, 4.19)	2.74 (0.18, 59.26)	FAR 6.0 Q8-Q16W	0.98 (0.16, 6.02)	0.65 (0.13, 3.49)	0.56 (0.08, 4.10)	0.86 (0.09, 12.72)	0.63 (0.12, 3.17)	**0.08 (0.01, 0.72)**
0.92 (0.28, 3.14)	1.05 (0.24, 5.06)	0.40 (0.04, 3.85)	2.82 (0.21, 58.12)	1.03 (0.17, 6.37)	RAN 0.5 PRN	0.66 (0.19, 2.63)	0.58 (0.09, 3.30)	0.89 (0.10, 10.39)	0.65 (0.15, 2.71)	**0.09 (0.02, 0.61)**
1.39 (0.54, 3.33)	1.59 (0.42, 5.85)	0.61 (0.07, 4.49)	4.24 (0.33, 69.87)	1.55 (0.29, 7.51)	1.51 (0.38, 5.34)	RAN 0.5 PRN load	0.87 (0.21, 3.15)	1.34 (0.16, 13.00)	0.99 (0.30, 2.72)	**0.13 (0.03, 0.71)**
1.60 (0.38, 7.06)	1.83 (0.33, 11.48)	0.70 (0.07, 6.49)	4.82 (0.32, 119.74)	1.78 (0.24, 13.24)	1.73 (0.30, 10.62)	1.14 (0.32, 4.80)	RAN 0.5 Q8W	1.52 (0.15, 24.23)	1.13 (0.28, 4.54)	0.15 (0.02, 1.37)
1.04 (0.12, 6.83)	1.19 (0.12, 9.87)	0.45 (0.02, 6.71)	3.14 (0.74, 15.04)	1.16 (0.08, 11.70)	1.13 (0.10, 10.25)	0.75 (0.08, 6.22)	0.66 (0.04, 6.89)	RAN 0.5 Q12W	0.74 (0.06, 5.77)	**0.10 (0.02, 0.43)**
1.41 (0.60, 3.76)	1.61 (0.46, 6.73)	0.62 (0.10, 3.58)	4.28 (0.36, 78.44)	1.58 (0.32, 8.28)	1.53 (0.37, 6.84)	1.01 (0.37, 3.31)	0.89 (0.22, 3.55)	1.35 (0.17, 15.65)	RAN 0.5 TREX	**0.13 (0.03, 0.82)**
**10.68 (2.45, 38.46)**	**12.17 (1.98, 60.12)**	4.66 (0.32, 46.17)	**32.49 (4.21, 268.35)**	**11.86 (1.40, 75.37)**	**11.59 (1.64, 61.65)**	**7.63 (1.42, 37.39)**	6.73 (0.73, 43.99)	**10.11 (2.32, 49.12)**	**7.60 (1.21, 34.79)**	SHAM

Key: AFLI – aflibercept; BEV – bevacizumab; CrI – credible interval; FAR – faricimab; OR – odds ratio; PRN – *pro re nata*; RAN – ranibizumab; TREX – treat and extend.

Note: Values presented are ORs and 95% CrI of the column intervention compared to the row intervention.

Table S7. Comparison of base case and sensitivity analysis removing studies with 100% Asian patients

	**Change from baseline in BCVA mean difference (95% CrI)**		**Proportion of patients gaining at least 15 letters OR (95% CrI)**		**Proportion of patients losing fewer than 15 letters OR (95% CrI)**	
Treatment	Base case	Sensitivity analysis	Base case	Sensitivity analysis	Base case	Sensitivity analysis
AFLI 2.0 PRN	-3.42 (-10.66, 3.84)					
AFLI 2.0 Q8W	-0.27 (-2.35, 1.80)	0.09 (-1.95, 2.40)	0.97 (0.67, 1.41)	0.97 (0.67, 1.45)	1.14 (0.44, 3.01)	1.14 (0.42, 3.31)
AFLI 2.0 TREX	-1.55 (-4.59, 1.53)	-1.47 (-4.65, 1.87)	0.77 (0.44, 1.37)	0.77 (0.42, 1.42)	0.44 (0.06, 2.95)	0.45 (0.06, 3.59)
BEV-g 1.25 Q4W	3.84 (-3.73, 11.46)	4.74 (-2.38, 11.94)	0.36 (0.07, 1.86)	0.36 (0.07, 1.91)	3.03 (0.29, 44.21)	3.00 (0.24, 40.84)
FAR 6.0 Q8-Q16W	0.09 (-2.92, 3.03)	0.50 (-2.42, 3.80)	1.04 (0.60, 1.84)	1.04 (0.59, 1.89)	1.11 (0.28, 4.28)	1.11 (0.25, 4.96)
FAR 6.0 Q12W	0.24 (-7.78, 8.19)	1.04 (-6.85, 8.90)	0.83 (0.19, 3.60)	0.83 (0.19, 3.68)		
FAR 6.0 Q16W	1.46 (-6.21, 9.15)	2.29 (-5.21, 9.87)	1.48 (0.39, 5.82)	1.48 (0.38, 5.98)		
RAN 0.5 PRN	-2.08 (-4.98, 0.68)	-1.81 (-4.72, 1.14)	**0.59 (0.35, 0.95)**	0.61 (0.37, 1.02)	1.08 (0.32, 3.53)	1.11 (0.31, 3.86)
RAN 0.5 PRN load	-1.43 (-3.38, 0.66)	-0.51 (-2.85, 2.27)	0.89 (0.64, 1.30)	1.04 (0.70, 1.68)	0.72 (0.30, 1.83)	0.82 (0.29, 2.67)
RAN 0.5 Q8W	-0.42 (-4.12, 3.40)	0.39 (-3.40, 4.45)	0.73 (0.38, 1.40)	0.81 (0.41, 1.63)	0.63 (0.14, 2.61)	0.68 (0.14, 3.27)

RAN 0.5 Q12W	-1.35 (-7.98, 5.27)	-0.59 (-6.72, 5.68)	**0.15 (0.04, 0.64)**	**0.15 (0.03, 0.67)**	0.96 (0.15, 8.41)	0.95 (0.13, 7.98)
RAN 0.5 TREX	0.24 (-1.62, 2.00)	0.48 (-1.37, 2.43)	1.00 (0.71, 1.39)	1.02 (0.72, 1.46)	0.71 (0.27, 1.66)	0.74 (0.28, 1.94)
SHAM	**-17.21 (-20.33, -13.86)**	**-17.01 (-20.12, -13.46)**	**0.10 (0.05, 0.22)**	**0.10 (0.05, 0.22)**	**0.09 (0.03, 0.41)**	**0.09 (0.02, 0.38)**

Key: AFLI – aflibercept; BCVA – best-corrected visual acuity; BEV – bevacizumab; CrI – credible interval; FAR – faricimab; OR – odds ratio; PRN – *pro re nata*; RAN – ranibizumab; TREX – treat and extend.

Note: Values presented are relative treatment effects compared to ranibizumab 0.5 mg every 4 weeks (reference treatment of network meta-analysis); all models are random effects models.

Table S8. SUCRA rankings of treatments in each analysis

Treatment	Change from baseline in BCVA at 12 months	Proportion of patients gaining at least 15 letters at 12 months	Proportion of patients losing less than 15 letters at 12 months
RAN 0.5 Q4W	0.634	0.725	0.607
AFLI 2.0 PRN	0.290	NA	NA
AFLI 2.0 Q8W	0.573	0.679	0.665
AFLI 2.0 TREX	0.376	0.485	0.293
BEV g 1.25 Q4W	0.863	0.295	0.879
FAR 6.0 Q8-Q16W	0.633	0.737	0.632
FAR 6.0 Q12W	0.589	0.562	NA
FAR 6.0 Q16W	0.696	0.820	NA
RAN 0.5 PRN	0.307	0.318	0.631
RAN 0.5 PRN load	0.378	0.599	0.421
RAN 0.5 Q8W	0.548	0.461	0.388
RAN 0.5 Q12W	0.442	0.078	0.548
RAN 0.5 TREX	0.671	0.715	0.414
SHAM	0.000	0.026	0.022
